# Coprological assessment of domestic carnivores in public areas and health education focused on larva migrans in São Paulo State, Brazil

**DOI:** 10.1590/S1984-29612025071

**Published:** 2025-12-08

**Authors:** Abraão Isaque da Silva, Adrieli Leite Araujo Moura, Beatriz Maia Galetti, Bruna Larocca Vieira, Camila Vitória Dias da Silva, Giovana Almeida Reis, Letícia Leite De Brito, Letícia Santos Goes, Luciana de Almeida Menezes, Matheus Porto Cortezi, Mayara Paula Paglione, Vanessa Kelen Nunes, Vitória Maximiana Soares dos Santos, Yasmin Saliba Maluf, Clara Morato Dias, Patricia Parreira Perin, Estevam Guilherme Lux Hoppe, Adolorata Aparecida Bianco Carvalho

**Affiliations:** 1 Universidade Estadual Paulista – UNESP, Faculdade de Ciências Agrárias e Veterinárias – FCAV, Departamento de Patologia, Reprodução e Saúde Única – DPRSU, Jaboticabal, SP, Brasil

**Keywords:** Cutaneous larva migrans, visceral larva migrans, helminth eggs, neglected zoonoses, Larva migrans cutânea, larva migrans visceral, ovos de helmintos, zoonoses negligenciadas

## Abstract

Cutaneous larva migrans (CLM) and visceral larva migrans (VLM) are neglected zoonoses caused by *Ancylostoma* spp. and *Toxocara* spp. Although investigations have been conducted in some areas of São Paulo State, Brazil, the presence of these helminth eggs in domestic carnivores’ feces from public areas in Jaboticabal (SP) had not previously been studied. This study aimed to detect *Ancylostoma* spp. and *Toxocara* spp. eggs in fecal samples collected in public squares using the Willis-Mollay method and to assess the community’s knowledge related to public health topics through a structured questionnaire. A total of 88 fecal samples were collected in 32 localities of the city, of which 12.5% (11/88) were positive for eggs of *Ancylostoma* spp. and none for *Toxocara* spp. The answers to the questionnaire revealed a direct trend between education level and knowledge of zoonotic risks, indicating that limited knowledge can be associated with a greater exposure to infection. This is the first study to report the presence of *Ancylostoma* spp. eggs in domestic carnivores’ feces in Jaboticabal. The results highlight the risk of CLM and reinforce the need for epidemiological surveillance in dogs and cats, along health education initiatives to promote regular deworming and the diagnostic testing of pets.

## Resumo

Larva migrans cutânea (LMC) e larva migrans visceral (LMV) são zoonoses negligenciadas causadas principalmente por *Ancylostoma* spp. e *Toxocara* spp. Até o momento, nenhum estudo relatou a presença de ovos de helmintos em fezes de carnívoros domésticos amostrados em áreas públicas da cidade de Jaboticabal (SP). Este estudo teve como objetivo detectar ovos de *Ancylostoma* spp. e *Toxocara* spp. em amostras fecais coletadas em praças públicas utilizando o método de flutuação de Willis-Mollay e avaliar o conhecimento da comunidade sobre temas relacionados à saúde pública por meio de um questionário. Foram coletadas 88 amostras fecais em 32 localidades do município, das quais 12,5% (11/88) foram positivas para ovos de *Ancylostoma* spp. e nenhuma para *Toxocara* spp. As respostas do questionário revelaram uma tendência direta entre escolaridade e conhecimento dos riscos zoonóticos, indicando que o conhecimento limitado pode estar associado a uma maior exposição à infecção. Relatamos, ineditamente, a presença de ovos de *Ancylostoma* spp. em fezes de carnívoros domésticos em Jaboticabal por meio de análises coproparasitológicas. Os resultados destacam o risco de LMC e reforçam a necessidade de vigilância epidemiológica aprimorada em carnívoros domésticos, juntamente com iniciativas de educação em saúde visando a desverminação e testes diagnósticos de animais de estimação.

## Introduction

Cutaneous larva migrans (CLM) and visceral larva migrans (VLM) are neglected zoonoses caused by gastrointestinal nematodes of dogs and cats, mainly affecting low-resource communities in South American and Caribbean countries. Both diseases are commonly associated with public environments, such as public squares, schools and playgrounds with sandpits, the circulation of stray animals and the population’s poor health education (Feldmeier, 2023). Children under ten years of age are the most affected by CLM and VLM in endemic areas (Jackson et al., 2006; Heukelbach et al., 2008; Feldmeier, 2023).

*Ancylostoma braziliense* and *A. caninum* are the most common species responsible for causing CLM. Human infection occurs by contact with contaminated soil with dog and cat feces, whose parasite penetrates the skin and migrates for several months in the epidermis, triggering intense inflammation associated with persistent itching (Heukelbach et al., 2003; Hoff et al., 2008; Del Giudice et al., 2019). Although it is a self-limiting disease, the infection may lead to sleep disturbance. Alternatively, *Toxocara canis* and *T. cati* are nematodes that cause VLM in humans. The infection occurs through the ingestion of eggs present in the feces of dogs and cats, causing a granulomatous reaction in the tissues in which their infective larvae have migrate, often being deposited in the liver, lungs, eyes, heart, and brain. Consequently, several clinical manifestations may occur, such as uveitis, chorioretinitis, Löffler syndrome, hepatosplenomegaly and less frequently cardiac, cerebral and muscular involvement (Hoff et al., 2008; Huynh et al., 2024). The adult forms of the nematodes that cause CLM and VLM live in the intestine of dogs and cats, and their eggs are eliminated in their hosts’ feces. The access of domestic animals to the streets, combined with the high number of stray animals in public areas and the lack or inefficiency of deworming practices, significantly increases environmental contamination with helminth eggs (Macpherson et al., 2023). The presence of eggs in the environment serves as a critical epidemiological marker for the potential transmission of these zoonotic parasites to human hosts (Błaszkowska et al., 2015; Reichert et al., 2016; Silva et al., 2020, Nezami et al., 2023; Macpherson et al., 2023).

Larva migrans reports in humans have been described in several countries, including Brazil (Jackson et al., 2006; Del Giudice et al., 2019; Reis et al., 2019; Rodrigues et al., 2019; Pourshahbazi et al., 2023). In France, Sow et al. (2017) reported CLM infections in travelers returning to the country between 2003 and 2015 through collected hospital data. In 37% of cases, the infection was acquired during travel to the Caribbean, Central America and South America; 33% in Africa; 28% in Asia; and 2% in Europe (Sow et al., 2017). The prevalence of *Ancylostoma* spp. eggs in dog and cat feces in Brazil varies regionally, with reported frequencies ranging from 45.6% to 73.7% in the northern parts of the country (Labruna et al., 2006; Monteiro et al., 2014; Corrêa et al., 2015; Mendonça et al., 2024); 43.3 to 96.8% in the Northeast (Campos et al., 2008; Ferreira et al., 2009; Ferreira et al., 2010; Lima et al., 2011; Silva et al., 2017); 29.5 to 84.4% in the Midwest (Almeida et al., 2007; Souza et al., 2023); 13 to 99% in the Southeast (Ribeiro et al., 2013; Moreira Alves et al., 2014; Curi et al., 2017; Santos et al., 2020) and 16.5 to 70.9% in the South (Blazius et al., 2005; Leite et al., 2007; Arruda et al., 2023). Regarding *Toxocara* spp., the prevalence is considerably lower, ranging from 1.2 to 30.7% throughout the Brazilian territory (Labruna et al., 2006; Leite et al., 2007; Ferreira et al., 2009; Ribeiro et al., 2013; Silva et al., 2017; Moreira Alves et al., 2014; Souza et al., 2023; Arruda et al., 2023; Mendonça et al., 2024). To date, there are no studies that aimed to detect such helminths in the feces of dogs and cats in the municipality of Jaboticabal, São Paulo state.

Poor health education has also been identified as a risk factor for humans acquiring infection by *Ancylostoma* spp. and *Toxocara* spp., especially in poor regions (Shimogawara et al., 2013; Reichert et al., 2016). Populations with low income and low educational levels tend to be unaware of zoonoses and preventive measures and have little access to basic sanitation services, which makes these communities the most vulnerable (Reichert et al., 2016; Martins-Melo et al., 2018; Feldmeier, 2023).

According to the Center for Social Policies – Getúlio Vargas Foundation (FGV, 2022) the state of São Paulo (SP) is among the ten Brazilian states whose population below the poverty line increased during the COVID-19 pandemic, favoring the occurrence of neglected zoonoses. Therefore, the present study aimed to detect eggs of *Ancylostoma* spp. and *Toxocara* spp. in fecal samples of domestic carnivores collected from the soil in public squares in the municipality of Jaboticabal, SP. Additionally, a questionnaire on health education was applied to the local population to estimate the risk of exposure to larva migrans, aiming at future strategies for preventive measures against zoonoses.

## Materials and Methods

### Study area

**Figure 1:**
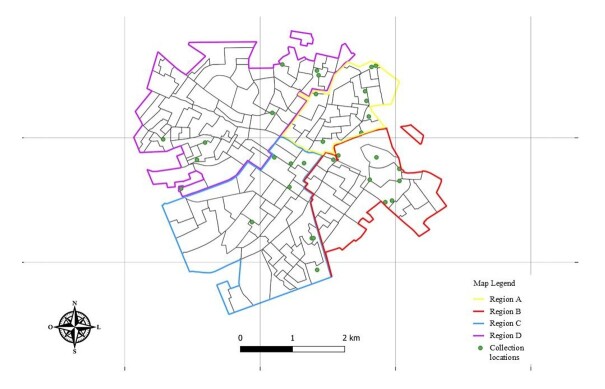
A representative map from Jaboticabal, SP, showing the sampling sites (green dots) in each city region (colored solid lines). QGIS Geographic Information System. Open Source Geospatial Foundation Project (QGIS, 2025).

Samples were collected from 32 public squares and parks located near residential areas, with the circulation of people and stray animals, in different regions of the municipality of Jaboticabal, São Paulo. For this purpose, the municipality was divided into four collection regions according to its geography and intramunicipal divisions, namely Region A, Region B, Region C, and Region D, all of them with eight sampling sites (Figure 1). The collections were carried out over a five-month period, from December 2022 to June 2023. The collection data were geoprocessed, associated with the presence or absence of parasites, and processed by the QGIS 2.18.2.8 software.

The city is located in the state’s northern region, with an estimated human population of 71,821 residents, according to the last demographic census in 2022. The Human Development Index (HDI) of this city was measured at 0.778 in 2010, and nearly 100% of the population has access to potable water and a sewage system (IBGE, 2022). As for its animal population, dogs are estimated at 16,083 and cats at 1,901 individuals (São Paulo, 2018).

### Sample collection and coprological tests

A total of 88 fecal samples were collected from domestic carnivores present in public squares and parks across all regions of the municipality of Jaboticabal, São Paulo, between December 2022 and June 2023. Fresh samples with little or no mucus were collected using procedure gloves and a sterilized spatula, stored in universal containers, labeled and refrigerated at 2–5 °C until processing at Laboratório de Enfermidades Parasitárias e Zoonoses (LabEPar) (UNESP/FCAV – Campus Jaboticabal, SP). The samples were processed by the Willis-Mollay flotation method. The feces are mixed with a saturated sodium chloride solution, allowing parasite eggs, which are lighter, to float to the surface. A coverslip is placed on the surface for a few minutes, then removed and examined under a microscope to identify and count the eggs.

### Health education assessment

During the collection period, a questionnaire was administered to individuals frequenting the sampling sites, covering topics such as zoonoses, knowledge about *larva migrans*, and environmental conditions in their community. The objective was to assess the population’s health education and the effectiveness of urban services and perform a descriptive analysis of the data collected from the interviewees. Additionally, the questionnaire responses and the occurrence of *Ancylostoma* spp. and *Toxocara* spp. eggs in the municipality of Jaboticabal, São Paulo, were discussed with respect to the convergence of findings, showing general trends observed in the results obtained.

The target population consisted of individuals frequenting public areas and squares in the municipality of Jaboticabal. Participants were selected through random sampling during their presence at the collection sites. They were invited to participate in the study via verbal invitations and completed a structured questionnaire guided by a researcher in a question-and-answer format (Appendix I). No inclusion or exclusion criteria were applied. A total of 48 individuals were approached, and all agreed to participate. All interviews were conducted within the same study area and concurrently with data collection.

### Statistical analysis

Statistical analysis was conducted using the Kruskal-Wallis test with Mann-Whitney-Wilcoxon post-hoc followed by Holm-Bonferroni correction to compare prevalence with the sampling region (A, B, C and D). The study was carried out using R software version 4.2.1 and the significance level adopted was 0,05.

## Results

### Coprological results

**Figure 2:**
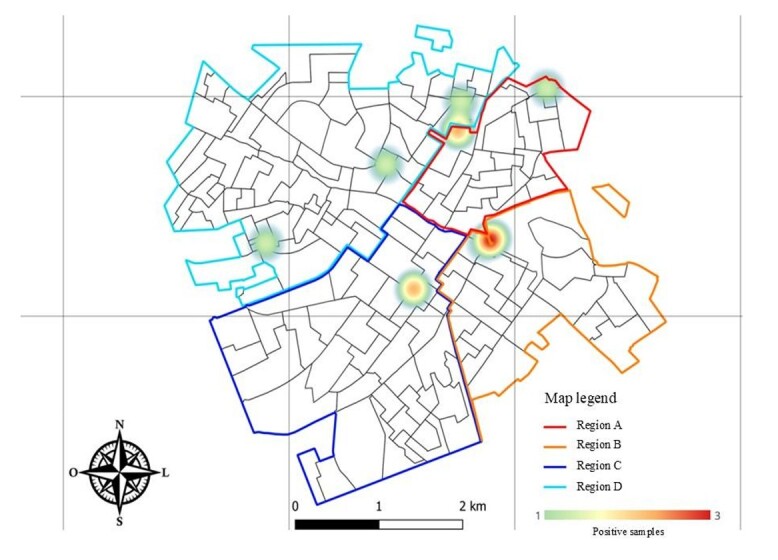
Heat map of *Ancylostoma* spp. samples positive from Jaboticabal city, SP. Red points indicate the presence of three positive samples within a 1-kilometer (km) radius, yellow points indicate two positive samples, and green points indicate one positive sample within the same radius QGIS Geographic Information System. Open Source Geospatial Foundation Project (QGIS, 2025).

The results for *Ancylostoma* spp. and *Toxocara* spp. in feces of carnivore domestics distributed in regions A, B, C and D of Jaboticabal city can be observed in Table 1 and Figure 2.

**Table 1:** Occurrence of hookworm and ascarid eggs in samples collected in regions A, B, C and D from Jaboticabal city, SP between July and December 2023. Statistical analysis was conducted using the Kruskal-Wallis test with Mann-Whitney-Wilcoxon post-hoc followed by Holm-Bonferroni correction to compare prevalence with the sampling region (A, B, C and D). The study was carried out using R software version 4.2.1 and the significance level adopted was 0.05.

**Region**		**A**	**B**	**C**	**D**	**Total**
**N samples**		**17**	**26**	**18**	**27**	**88**
***Ancylostoma* spp.**	**N positive samples**	3	3	2	3	11
	**Frequency of positive samples by region (%)**	17.60%	11.50%	11.10%	11.10%	12.50%
	**Standard deviation**	0.393	0.326	0.323	0.320	0.330
	**CI**	(3.80 – 43.43%)	(2.40 – 30.15%)	(1.38 – 34.71%)	(2.35 – 29.16%)	(7.1 - 21.0%)
***Toxocara* spp.**	**N positive samples**	0	0	0	0	0
	**Frequency of positive samples by region (%)**	0%	0%	0%	0%	0%

N: Absolute number of data; CI: Confidence interval.

Positive samples for *Ancylostoma* spp. obtained from different regions of the municipality were represented on a Kernel density map (Figure 2). Red points indicate the presence of three positive samples within a 1-kilometer (km) radius, yellow points indicate two positive samples, and green points indicate one positive sample within the same radius. Region A showed a positivity of 17.60% (3/17), with one sample at coordinate 21°14'22.8”S 48°18'18.2”W and two samples at 21°14'35.9”S 48°18'49.4”W. Region B showed a positivity of 11.50% (3/26), with two samples at coordinate 21°15'16.6”S 48°18'35.0”W and one at 21°15'21.1”S 48°18'41.7”W. A positivity of 11.10% (2/18) was found in Region C at coordinate 21°15'37.9”S 48°19'10.3”W, and 11.10% (3/27) in Region D at coordinates 21°14'50.5”S 48°19'21.9”W, 21°15'21.7”S 48°20'12.0”W, and 21°14'25.6”S 48°18'51.2”W.

### Health education assessment and urban services effectiveness based on the questionnaire applied to the local community

Although 83.34% (40/48) of the residents stated that dogs and cats can transmit diseases to humans, only one participant (2.08%, 1/48) was able to explain the concept of zoonoses. Among the interviewees, 37.5% (18/48) correctly cited possible diseases transmitted by animals, with toxoplasmosis and rabies being the most cited. Furthermore, when asked about cutaneous larva migrans, 27.08% (13/48) stated that they knew about “*bicho geográfico*”, a name popularly used to refer to the disease in Brazil.

Regarding the prevention of VLM and CLM, only 14.58% (7/48) of the interviewees reported that avoiding contact with animal feces, as well as food and hand washing, are ways to prevent these parasitic infections. Additionally, 8.34% (4/48) of the interviewees declared that they knew about *Toxocara* spp. or VLM; however, none of them knew about forms of transmission.

As for the personal data of the interviewees, 20.83% (10/48) declared having completed higher education, 33.34% (16/48) had completed high school and 16.67% (8/48) had completed elementary school. Still, 25% (12/48) of the interviewees stated that they had not completed elementary school and 4.1% (2/48) did not wish to declare their level of education. In addition, among the occupations of the interviewees, 12.5% (6/48) were students, 6.25% (3/48) were unemployed, 8.33% (4/48) were retired, 2.08% (1/48) were government employees and 70.83% (34/48) worked as freelancers. None of them chose to report on their family income.

In what concerns the interviewees’ residence, 75% (36/48) stated the presence of parks and/or public squares close to their homes. When asked about the existence of dog and cat deworming campaigns in the municipality, 25% (12/48) of the interviewees answered affirmatively and 75% (36/48) said they were not aware of this activity. When the interviewees were asked about their knowledge of measures taken by public agencies to reduce the population of stray dogs and cats, 35.41% (17/48) of the individuals stated that they were aware of some measure. On the other hand, 64.58% (31/48) said they were not aware and/or were not aware of such measures in the municipality. Among the measures mentioned by the interviewees, neutering projects stand out. All 100% (48/48) stated that they had access to water supply and sewage services in their homes, in addition to living in neighborhoods with paved streets.

## Discussion

In the present study, coproparasitological tests were used to detect the occurrence of *Ancylostoma* spp. and *Toxocara* spp. eggs in domestic carnivores feces from 32 areas of public squares and parks selected in Jaboticabal city, SP. Among the 88 samples collected and submitted to Willis-Mollay flotation method, 12.5% (11/88) of *Ancylostoma* spp. eggs and no *Toxocara* spp. eggs in domestic carnivores feces were detected. Although studies have been carried out in some municipalities in the state of São Paulo, aiming to detect these parasites in the feces of domestic carnivores (Moreira Alves et al., 2014; Silva et al., 2020; Santos et al., 2020), no study investigated helminth eggs in feces of domestic carnivores sampled in Jaboticabal’s public areas, making this the fir study to describe the occurrence of such agents in the aforementioned city, to the best of our knowledge.

The frequency detected in the present study corroborates findings from other Brazilian states in which *Ancylostoma* spp. was the nematode most frequently detected, followed by *Toxocara* spp. (Moreira Alves et al., 2014; Curi et al., 2017; Guimarães et al., 2019; Ramos et al., 2020; Arruda et al., 2023; Souza et al., 2023). In Botucatu (SP), Santos et al. (2020) reported a 12.1% frequency of *Ancylostoma* spp. eggs in naturally infected dogs, with 17.6% showing co-infection with *Toxocara* spp. Similarly, Moreira Alves et al. (2014) found 23.4% positivity for *Ancylostoma* spp. and 8.1% for *Toxocara* spp. in 640 fecal samples from dogs in public squares in Pindamonhangaba (SP). Comparable results were also observed by Arruda et al. (2023), who detected *Ancylostoma* spp. in 16.5% and *Toxocara* spp. in 5.5% of 91 fecal samples from rural dogs in Painel (SC), results consistent with the present study. Regarding statistical analysis, there was no correlation between sample positivity and sampled region, requiring a larger sampling of domestic carnivores feces in future studies to assess the vulnerability of such regions to the studied parasites.

The Willis-Mollay flotation technique (Ueno & Gonçalves, 1998) used in the present study continues to be a rapid and inexpensive analysis for the diagnosis of soil-transmitted helminths. Macpherson et al. (2023) compared the results obtained in the detection of helminth eggs through the flotation and multiplex qPCR techniques in 211 fecal samples from dogs and found similar results for both diagnostic methods. In the multiplex qPCR assays, the aforementioned study detected 40.8% (86/211) of samples positive for *Ancylostoma* spp. and 5.7% (12/211) for *T. canis*, while in the flotation technique, the frequencies found for the same helminths corresponded to 46.5% (98/211) and 5.2% (10/211), respectively. One important and internationally recognized indicator of infection by such zoonotic agents is the detection of eggs in the external environment (Błaszkowska et al., 2015; Reichert et al., 2016; Silva et al., 2020; Nezami et al., 2023; Macpherson et al., 2023).

Although Jaboticabal city has favorable climatic conditions for the development and persistence of *Ancylostoma* spp. and *Toxocara* spp. in the environment, the frequencies detected for helminths were considered below expectations, considering the relevant number of animals with easy access to the streets and non-domiciled animals circulating in the municipality. These results may be related to the parasite’s biology, as the larvae of *Toxocara* spp. tend to enter a state of hypobiosis in the tissues of dogs older than four months and do not complete their development, making it impossible to eliminate eggs in the host’s feces (Taylor et al., 2017). Therefore, the absence of *Toxocara* spp. eggs detected in the present study does not rule out the hypothesis that dogs and residents of Jaboticabal are exposed and susceptible to this pathogen. Another possibility may be related to the type of sample chosen for the study, requiring future analyses with soil samples from public areas where domestic carnivores’ feces are present.

The exposure of humans to public environments contaminated with eggs of *Ancylostoma* spp. and *Toxocara* spp. increases the risk of infection by these causative agents of CLM and VLM, requiring a One Health approach for integrated surveillance between humans, animals, and the environment, considering the zoonotic nature of such diseases. In Brazil, soil-transmitted helminthoses, including ascaridosis, trichurosis, and hookworm infection, are part of the group of neglected tropical diseases (NTDs) that primarily affect children under ten years old in areas where sanitary conditions lack (Feldmeier, 2023; Martins-Melo et al., 2018). Poor health education in urban environments and failures in responsible care by pet owners are important risk factors for the development of larva migrans in humans. Nunes & Rocha (2019) showed that the presence of parasites in adolescents in a slum in Maceió, Alagoas state, occurred due to the lack of basic sanitary conditions associated with the presence of pet feces in the environment. Regions marked by inefficiency in water supply, sewage collection and treatment, urban cleaning with garbage collection and disposal, as well as drainage and management of rainwater, become favorable places to the spread of parasitic diseases, such infections constituting an important socioeconomic indicator (Souza et al., 2023; Nunes & Rocha, 2019; Silva et al., 2015). In the present study, although all individuals interviewed stated that they lived in places where basic sanitation was provided by the municipal administration and 83.34% (40/48) stated that they understood what zoonoses are, only 2.08% (1/48) of the interviewees were able to explain and exemplify zoonotic diseases.

Likewise, the understanding of zoonoses, the helminths that cause larva migrans, the forms of transmission, and prophylactic measures were proportionally related to the interviewee’s level of education. The higher the participants’ educational level, the greater their knowledge regarding such helminthosis. Data from the interview reveal that all individuals with incomplete elementary education were unaware both of CLM and/or VLM and of how to prevent infection by the parasites in animals and humans; the opposite occurred among interviewees with a higher education degree, showing that education is also an important factor in these parasitic infections’ prevention.

Among important prophylactic measures against larva migrans, hand washing is essential in preventing soil contamination with helminths and the transmission of agents (Macpherson et al., 2023; Walker et al., 2023). Deworming companion animals is also an important strategy to prevent the transmission of such helminths and should be carried out cautiously and under the supervision of a veterinarian. Resistance to anthelmintics due to indiscriminate use has been reported in helminths of the genus *Ancylostoma* in Canada (Nezami et al., 2023), the USA (Jimenez Castro et al., 2019, 2021), Nigeria (Jimenez Castro et al., 2020), Australia (Kopp et al., 2008), and Brazil (Furtado et al., 2014; Silva Medeiros et al., 2022). In Brazil, Furtado et al. (2014) detected F200Y SNP in the *b-tubulin* gene associated with benzimidazole resistance in *Ancylostoma caninum* isolated from fecal samples of dogs in the states of Minas Gerais and Piauí. Resistant helminth strains may be related to the worsening of parasitic diseases, requiring recurrent treatment, and reinfections of humans in environments with poor sanitation due to exposure to environmental parasite load.

The health education approach allows for the sharing of diverse knowledge to face challenges such as the diseases addressed in this study, which are widely distributed and closely linked to socioeconomic issues and poor sanitation conditions, especially impacting vulnerable communities (Albuquerque et al., 2013). Actions such as lectures, the distribution of information leaflets, social media campaigns on zoonoses, responsible ownership, and adequate deworming after coprological analyses are important means of raising awareness among the population. Good health education associated with the strengthening of epidemiological surveillance should enable the reduction of cases in areas of greater social vulnerability, mitigating their impacts on human health.

## Conclusion

This study reports for the first time the coprological detection of *Ancylostoma* spp. eggs in domestic carnivores’ feces in the municipality of Jaboticabal, SP, as well as a deficient understanding of zoonoses and prophylactic measures among the interviewed population of the city, particularly those with low educational levels.

## Supplementary Material

Supplementary material accompanies this paper.

Appendix I Questionnaire for the community of Jaboticabal – SP

This material is available as part of the online article from https://doi.org/10.1590/S1984-29612025071
