# Supplementary material for: Coprological assessment of domestic carnivores in public areas and health education focused on larva migrans in São Paulo State, Brazil
Source: Rev Bras Parasitol Vet. 2025 Dec 8;34(4):e009925. doi: 10.1590/S1984-29612025071 (PMC12704777; doi:10.1590/S1984-29612025071)
Supplement: Appendix I [file rbpv-34-4-e009925-suppl01.pdf]

APPENDIX I – Questionnaire for the community of Jaboticabal – SP

DATE: ____/____/____ FILE Nº ___________________________

NAME:____

PROFESSION:__________________

PHONE:_______________________________

E-MAIL:______________________________

ADDRESS:____

EDUCATION LEVEL:_________________________

Data on general knowledge of human and animal health

1. **Explain what zoonoses are? __________________.**
2. **Do you think dogs can transmit any disease to humans? ( )No ( )Yes. If yes, which ones? Please name two?__________________________________.**
3. **Do you have knowledge about geophagous parasites (geohelminths)? ( )No ( )Yes If yes, provide two pieces of information: __________________________________.**
4. **If so, do you know how they are transmitted?**
5. **If so, list some measures that you consider important to prevent this disease.**
6. **Do you have knowledge on Toxocara spp. or larvae migrans visceral? ( )No ( )Yes If yes, provide two pieces of information __________________________________.**
7. **If so, do you know how it is transmitted?**
8. **If so, name some measures that you consider important to prevent this disease.**
9. **If your dog or cat has any of these diseases:**

( )We should do nothing because the disease heals itself

( )We should not do anything because the disease is not bad for us

( )We should give dewormers indicated by the veterinarian

( )We should give antibiotics to the animals

( )Other _____________________

Environmental situation data

10. **Is the street where you live paved? ( )Yes ( )No.**

11. **Does it have a sewage system? ( )Yes ( )No. If not, indicate the type: Septic tank ( ) Open sky ( ) Other ________________________**

12. **Is the water treated? ( ) Yes ( ) No ( )Well**

13. **Are there parks or public recreation areas in your neighborhood? ( ) Yes ( ) No**

14. **If so, do you visit these environments with your animals? ( ) Yes ( ) No**

15. **Do the municipality's public agencies have measures to control the population of stray dogs and cats? ( ) Yes ( ) No**

16. **Are there dog and cat deworming campaigns in the municipality? ( )Yes ( ) No**

17. **Do the neighborhoods of the students who live in the school have basic sanitation?**
